# Maternal Diet and Nutrient Requirements in Pregnancy and Breastfeeding. An Italian Consensus Document

**DOI:** 10.3390/nu8100629

**Published:** 2016-10-14

**Authors:** Franca Marangoni, Irene Cetin, Elvira Verduci, Giuseppe Canzone, Marcello Giovannini, Paolo Scollo, Giovanni Corsello, Andrea Poli

**Affiliations:** 1 NFI—Nutrition Foundation of Italy, Milano 20124, Italy; poli@nutrition-foundation.it; 2 Department of Mother and Child Hospital Luigi Sacco, Center for Fetal Research Giorgio Pardi, Department of Biomedical and Clinical Sciences University of Milan—Italian Society of Perinatal Medicine (SIMP), Milano 20157, Italy; irene.cetin@unimi.it; 3 Department of Pediatrics, Department of Health Sciences, San Paolo Hospital, University of Milan—Italian Society of Pediatrics (SIP), Milano 20142, Italy; elvira.verduci@unimi.it; 4 Obstetrics and Gynecology Unit, S. Cimino Hospital—Italian Society of Gynecology and Obstetrics (SIGO), Termini Imerese, Palermo 90018, Italy; gicanzone@tiscali.it; 5 Department of Pediatrics, San Paolo Hospital, Department of Health Science, University of Milan—Italian Society of Pediatric Nutrition (SINUPE), Milano 20142, Italy; marcello.giovannini@unimi.it; 6 Division of Gynecology and Obstetrics, Maternal and Child Department, Cannizzaro Hospital—Italian Society of Gynecology and Obstetrics (SIGO), Catania 95126, Italy; presidente@sigo.it; 7 Department of Sciences for Health Promotion and Mother and Child Care, University of Palermo—Italian Society of Pediatrics (SIP), Palermo 90127, Italy; giocors@alice.it

**Keywords:** nutrient requirement, pregnancy, breastfeeding, adequate intake, DHA, micronutrients

## Abstract

The importance of lifestyle and dietary habits during pregnancy and breastfeeding, for health of mothers and their offspring, is widely supported by the most recent scientific literature. The consumption of a varied and balanced diet from the preconceptional period is essential to ensure both maternal well-being and pregnancy outcomes. However, the risk of inadequate intakes of specific micronutrients in pregnancy and lactation is high even in the most industrialized countries. This particularly applies to docosahexaenoic acid (DHA), iron, iodine, calcium, folic acid, and vitamin D, also in the Italian population. Moreover, the risk of not reaching the adequate nutrient supply is increased for selected groups of women of childbearing age: those following exclusion diets, underweight or overweight/obese, smokers, adolescents, mothers who have had multiple or close pregnancies, and those with previous unfavorable pregnancy outcomes.

## 1. Introduction

Diet and lifestyle are important determinants of health of both mother and offspring, starting from the preconceptional period.

In particular, current research underscores that the first 1000 days of life (from conception up to two years of life) are crucial for the prevention of adulthood diseases [1] and that specific maternal conditions during the periconceptional period (particularly obesity and excessive weight gain during pregnancy) are associated with high birth weight, obesity and alterations in glucose metabolism in children and, later, in adults, with increased cardiometabolic risk [2].

Health later in life is also affected by the growth rate in the first months of life, when breastmilk represents the gold standard for infant feeding, as recognized by the WHO, who defines it part of the reproductive process, with important implications also for the health of lactating mothers, ranging from the reduction of cardiovascular risk and hip fractures in post-menopause, to protection against some types of cancers, such as breast and ovarian cancers. In this regard, the World Cancer Research Fund includes breastfeeding among its 10 recommendations aimed at cancer prevention [3].

Indeed, exclusive breastfeeding is recommended for at least six months by the Italian Position Statement on Breastfeeding and use of maternal/human milk is strongly supported by a document, recently published by five national medical and scientific societies, who agree on its socio-economic consequences on families and society and on the need for careful evaluation of possible contraindications, according to evidence-based criteria [4].

The aim of this consensus document is to review the available literature on dietary requirements and health of women during pregnancy and breastfeeding, focussing on selected nutrients for which the need of greater attention is supported by solid evidence, both in the general female population, and in specific population groups.

## 2. Energy and Macronutrients

The caloric requirements for healthy, normal weight women with a moderately active lifestyle, undergoes a moderate increase during pregnancy (dependent on pregnancy stage), which can be met by slightly increasing energy intakes, in a balanced equilibrium between macronutrients within the recommendations of nutritional guidelines. Excess of calories and macronutrients during pregnancy may, in fact, be just as damaging as their deficiency, especially in overweight and obese women, with an increased risk of miscarriage, gestational diabetes, pre-eclampsia and also of obesity and type 2 diabetes for their children in adulthood [5,6]. In addition, during lactation only a moderate increase in the mother’s energy needs is necessary for milk production.

The 2014 Italian RDA [7], specifically, indicate an additional requirement of 69 kcal/d for the first trimester, 266 kcal/day for the second and 496 kcal/day in the third trimester of pregnancy (for a grand total of an additional 76,530 kcal). Very similar amounts have been established by the EFSA (70 kcal/day in the first trimester to 260 and 500 kcal/day in the second and third, respectively), with an increase of about 500 kcal/day during the first 6 months of exclusive breastfeeding [8].

### 2.1. Protein

Among the macronutrients, protein requires more attention during pregnancy, when demand progressively increases to support protein synthesis, in order to maintain maternal tissues and fetal growth, especially during the third trimester. An excessively low intake of protein is associated with potentially negative effects in terms of weight and length at birth; on the other hand, an excessively high proportion of protein could affect fetal development [9].

The protein quality of foods is measured by their PDCAAS (Protein Digestibility Corrected Amino Acid Score), which is the score for amino acid digestibility [10]. Values close to 1 are typical of animal products, providing all nine essential amino acids, while values below 0.7 are typical of plant products. However, the consumption of two or more vegetable foods with different amino acidic composition can help improving the overall quality of their protein component [11].

International guidelines agree in recommending and increased protein intake during pregnancy, especially during the second and third trimesters to ensure the additional 21 grams needed for maternal and fetal tissues and placenta [12,7]. In this regard, the recommended daily allowances (defined as PRI-population reference intake: the dietary requirement at the 97.5th percentile) should be increased by 1 g/day in the first trimester of gestation, 8 g/day in the second trimester, and 26 g/day in the third trimester [7].

Also during exclusive breastfeeding, habitual protein intake should be increased by 21 g/day in the first semester and 14 g/day later, if breastmilk still represents a substantial proportion of the infant’s diet [7].

### 2.2. Fat

During pregnancy, the quality of fats is more important than their total amount, especially for fetal development and infant growth. For this reason, it is necessary to improve the relative proportion of polyunsaturated fats rather than to increase the intake of total fats: an adequate intake of docosahexaenoic acid (DHA, of the *n*-3 series), essential for the growth and development of brain and retina, is of utmost importance.

After delivery, breastmilk fat content is mainly dependent on the feeding period, the stage of the feed, and the number of pregnancies, while maternal diet (energy intake, amount of dietary fat) and lifestyle are less relevant (except in cases of severe malnutrition) [13]. In fact, the release of deposits in the maternal compartment reflects the long-term food intake. During pregnancy and lactation it is thus not necessary to change the overall fat intake [14].

### 2.3. DHA

DHA is the major polyunsaturated fatty acid contained in the human brain and retinal rods and, thus, is essential for brain and retinal development of the fetus during pregnancy. In fact, DHA plays major roles in the psychomotor neurodevelopment in the first months of life, when it is supplied at high amounts by breastmilk (while, as an example, it is not contained in cow’s milk) [15,16].

The benefits of DHA for the fetus and for the infant are supported by an extensive literature that confirms the importance of appropriate omega-3 intake for maternal health (to reduce the risk of premature birth and post-partum depression), for the composition of breastmilk, and for overall infant health [17,18,19].

Even though the human body holds the necessary enzymatic pathway for the synthesis of DHA (the *n*-3 fatty acid with the longest carbon chain and higher degree of unsaturation) from the metabolic precursor (i.e., alpha-linolenic acid or ALA), there is clear experimental evidence that the conversion of ALA to longer chain fatty acids is quantitatively insufficient to ensure adequate tissue levels. In fact, it has been demonstrated that the efficiency of the conversion of ALA to EPA (eicosapentaenoic acid) is highly variable, but lower than 10%; the conversion of ALA to DHA is even lower and virtually nil in male subjects [20,21].

Due to this limited ability of the human body to synthesize long chain polyunsaturated fatty acids, in recent years the concept of essentiality was extended from ALA (which is present in high concentrations in some vegetable oils and is a minor component of almost all plants) to EPA and DHA, which are contained in high concentrations only in fatty fish living in cold seas (mackerel, anchovies, and salmon), with a high variable ratio of EPA to DHA (Table 1) [22].

Foods of terrestrial origin can only minimally contribute to long chain *n*-3 intake. Therefore, diets devoid of fish (such as the typical Western diet) are mostly deficient in DHA as well as EPA. It is estimated that insufficient intakes of long-chain omega-3 in the diet comes in second (after the high consumption of salt) among the causes of diet-related mortality in the United States [24]. There is some evidence that approximately 80% of the population (also in Italy) does not ingest the daily amount of EPA and DHA recommended by international guidelines (250–500 mg daily) [25,26].

Due to concerns of the risk of contaminants in fish, a recent opinion of the European Food Safety Authority indicates that consuming 1–2 up to 3–4 servings of fish per week during pregnancy ensures proper development of the offspring, and it emphasizes that, at least in Europe, these levels of consumption are not associated with significant risk in terms of contamination by methyl-mercury [27]. The EFSA report concludes that consumption of more than 3–4 servings of fish/week does not provide any additional benefit. In order to balance adequate amounts of EPA and DHA and lower the risk of environmental contaminants, smallest fish such as sardines, anchovies and mackerel should be preferred [22].

Recently, it has been possible to obtain from the primary source of long-chain *n*-3 fatty acids, i.e., algae, more purified safe formulations that predominantly contain bioavailable DHA and that, unlike fish oils, are odorless and can be more easily integrated in food [28].

According to EFSA and the Italian RDA [7], the DHA requirement increases to 100–200 mg per day during pregnancy and lactation, based on studies that have shown the association between increase in the content of DHA in breastmilk and a better overall health status of the infant, especially in terms of visual acuity and cognitive development. The consumption of two servings of fish per week allows achieving the adequate DHA content in breastmilk [14].

Excessively low blood concentrations of DHA have been in fact reported in women following exclusively vegetarian diets or who do not have an adequate intake of fish [29]. In addition, special population groups, such as mothers who keep smoking during pregnancy or during lactation might require more DHA: infants born to women who smoke are smaller for gestational age at birth and show significantly reduced circulating concentrations of DHA compared to those born to non-smoking mothers [30]. In the postnatal period, instead, maternal smoking has been linked with a low provision of DHA with breastmilk, to the newborn [31].

## 3. Micronutrients

During pregnancy, micronutrient requirements increase more than those of macronutrients, and inadequate intakes (and, thus, a low nutritional quality of the diet) can have significant consequences for both the mother and the developing fetus. In particular, there is evidence to support the physiologic role played by selected minerals and vitamins [12,32].

### 3.1. Iron

Involved in numerous enzymatic processes, iron (the foremost constituent of hemoglobin, myoglobin and various enzymes) plays essential roles in the transfer of oxygen to tissues. Iron deficiency causes anemia, a very common condition worldwide, affecting 22% of women of childbearing age in Europe and as much as 50% in developing countries [33]. In addition, iron deficiency is frequent in children between 6 and 36 months of age [34].

Meat and fish, but also legumes and green leafy vegetables are the main dietary sources of iron. In Italy, most dietary iron is found in a non-heme form, the absorption of which is closely linked to the overall composition of the diet and the individual nutritional status. For example, phytates and polyphenols are able to inhibit the absorption of non-heme iron, which is favored by ascorbic acid or by the consumption of meat and fish. In general, the human body is able to absorb 2%–13% of non-heme versus about 25% of heme iron [7].

During pregnancy, iron requirement progressively increases until the third month, in parallel with the accumulation in fetal tissues. The transfer from the maternal compartment to the fetus is regulated by a complex mechanism of transport that include: release from maternal liver—in which it is stored as ferritin—into circulation as Fe^2+^, uptake by the placenta, transfer to the fetus (by a specific protein), oxidation to Fe^3+^, storage (as ferritin) or transport into the fetal circulation (still bound to transferrin) [35].

Inadequate intakes during pregnancy associated with the increase of iron demand makes pregnant mothers at even greater risk of iron deficiency, that may affect growth and development of the fetus and increase the risk of preterm delivery, low birth weight and post-partum hemorrhages [36,37]. Moreover, according to some recent studies, inadequate iron intakes during pregnancy are associated with increased cardiovascular risk for the offspring in adulthood [38].

In fact, iron supplementation in pregnancy is often recommended to improve pregnancy and birth outcomes [35,37,39]. On the other hand, an excessively high iron intake may expose women to oxidative stress, lipid peroxidation, impaired glucose metabolism, and gestational hypertension [40]. International recommendations in terms of intake levels range from the 27 mg per day for all pregnant women as advised by the Center for Disease Control and Prevention and the WHO to the 30–60 mg as advised by the Italian RDA (Table 2).

The immediate postpartum period is characterized by maternal susceptibility to anemia because of blood loss at delivery even in industrialized countries, where almost 50% of women require iron supplementation. However, the amount of iron secreted in milk is quite small and the WHO and FAO indications support a reduced supply of iron during breastfeeding to compensate for amenorrhea. Eleven mg per day should therefore be recommended and increased to 18 mg/day after the resumption of menstruation.

### 3.2. Iodine

Iodine is a major component of thyroid hormones and is essential for their functions, namely growth, formation and development of organs and tissues, in addition to the metabolism of glucose, proteins, lipids, calcium and phosphorus, and thermogenesis. Iodine is mostly found in organic form in the body, bound to thyroglobulin. The inadequate availability of iodine causes deficiency of circulating thyroid hormones, increase of pituitary thyroid stimulating hormone (TSH) and the consequent hypertrophy of the thyroid gland (goiter) [42].

Fish and shellfish are the main food sources of iodine, receiving it from the algae they eat, that absorb the mineral from marine water. However, due to water evaporation and rain, iodine is also absorbed by the soil and, consequently, enters into water, fruits, vegetables, and—in relevant concentrations—in milk, eggs and then meat (to a variable extent).

The average daily intake of iodine in the general population is less than that indicated by WHO, at the European level, where iodine deficiency affects mainly the child population [43], and all over the Italian territory (85–88 µg/day vs. 150 µg/day) [44].

In pregnancy, iodine deficiency can increase the risk of spontaneous abortion, perinatal mortality, birth defects and neurological disorders [45], and is considered by the WHO as the most important preventable cause of brain damage.

In the general population, iodine deficiency can be prevented by supplementing the diet with adequate amounts of this mineral, for example by using iodized salt.

During pregnancy, when iodine is necessary also for the production of fetal thyroid hormones (as the fetal thyroid begins to function only around the twelfth week of gestation), women need to increase iodine intake by about 50% [46,47].

Moreover, even in conditions of only mild or moderate iodine nutritional deficiency, the fetus and the newborn (especially preterm born) have a much higher risk of developing hypothyroidism compared to all other age groups (National Observatory for the Monitoring of Iodoprophylaxis in Italy). The most critical period goes from the second trimester of pregnancy to the third year of extrauterine life. Adequate supplementation with iodine, from pre-conception and until the end of the first trimester of pregnancy, reduces—up to 73%—the incidence of cretinism in the areas of highest deficiency risk [48]. The estimated amount that would avoid deficiency is 200 µg/day (compared to 150 µg/day for adults) according to the EFSA, or 250 µg/day according to the WHO/UNICEF joint document [49]. Two hundred µg/day are recommended also during lactation, to ensure a milk content of about 100–150 µg/100 mL.

### 3.3. Calcium

As the most abundant mineral in the human body, 99% located in the skeleton and in the teeth, calcium is critical to reach the peak bone mass in the first decades of life, to maintain bone mass in adulthood, and to slow the physiological age related reduction of bone mineral density.

Calcium deficiency may be worsened by genetic and hormonal factors along with insufficient physical activity. Calcium metabolism also requires vitamin D, the lack of which can also be due to calcium deficiency: in both cases, the result is a reduced mineralization of the bone matrix. Inadequate levels of calcium in children can result in rickets [50].

The main sources of calcium are milk and derivatives (about 50%), followed by cereals and vegetables (11% each) [51]. The bioavailability of calcium from these foods is different, being highest for milk and derivatives and for mineral water. Conversely, bioavailability from fiber- and phytate-rich vegetables is quite low. The efficiency of calcium absorption from food affects calcium concentrations in the body, which remains constant from adolescence to adulthood and decreases in post-menopausal women, by 2% every 10 years [50].

The EPIC (European Prospective Investigation into Cancer and Nutrition) study has shown a wide variability in calcium levels among the different European populations, with the lowest values in Italian women [52]. According to the results of the Italian survey INRAN-SCAI 2005-06, calcium intake in the Italian population corresponds to 76% of the recommendations [53].

Calcium is essential for fetal development. The requirement increases during pregnancy (from 50 mg/day at the halfway point, up to 330 mg/day at the end) and lactation, due to the mobilization from the maternal skeleton, the greater efficiency of intestinal absorption and the increased renal retention [54]. High birth weight, reduced risk of preterm delivery, and better blood pressure control are also associated with an adequate calcium intake during pregnancy. The transport of calcium from the maternal compartment to the fetus takes place through active transporters in the epithelial layer of the placenta. From the 20th week of pregnancy, calcium levels in the fetal circulation are higher than those detectable in the maternal plasma. 

The recommendations for calcium intake are different in different countries, also for pregnant and breastfeeding women. The Italian RDA indicate PRI values of 1.2 g/day in the gestational period, while the WHO recommends 1.5–2.0 g/day from the 20th week until the end of pregnancy, especially for women at risk of gestational hypertension.

It has been proposed that a low-dose supplementation with calcium during pregnancy reduces the risk of developing both gestational hypertension and pre-eclampsia [55]. However, excessively high levels correlate with increased risk of developing HELLP (Haemolysis, Elevated Liver enzymes and Low Platelets) syndrome.

The daily amount of calcium secreted in breastmilk is quite variable (150 to 300 mg/day), mainly depending on the mobilization from bones and the reduced urinary secretion. Calcium stores in maternal bones are restored after weaning and the recovery of ovarian function [56].

Some studies have shown that calcium secretion in milk is substantially independent of its dietary intake and of supplementation. Therefore, the recommended intake during lactation is not different from that of the healthy adult female population (1.0 g/day). However, women with dietary calcium intakes lower than 300 mg/day and adolescents, with high basal requirements (1.2 g/day according to the RDA) are at risk of deficiency also during lactation.

### 3.4. Vitamin D

The term vitamin D comprises the two main molecular species that share vitamin activity: cholecalciferol (vitamin D3, derived from cholesterol and synthesized by the animal organisms) and ergocalciferol (vitamin D2, derived from ergosterol, found in vegetables). 

The circulating levels of vitamin D are only partly affected by the dietary intakes. In fact, only the first of the two hydroxylation processes occurring in vitamin D metabolism (e.g., that responsible for the production of 25-hydroxy-vitamin D) is modulated by the dietary contribution to some extent (the increase of circulating levels is not proportional to the amount ingested). The hydroxylation into 1,25-hydroxy-vitamin D in the proximal renal tubules is closely regulated by feedback mechanisms and primarily depends on the requirement for calcium and phosphorus [57].

The endogenous synthesis of vitamin D requires exposure to ultraviolet radiation with a wavelength between 290 and 315 nm, and is influenced by several factors, related to both the individual’s characteristics (such as sex and phenotype, weight), and environmental factors (the degree of physical activity, latitude, season, time of exposure to sunlight, pollution, use of sunscreens and supplements). With aging, the synthesis of vitamin D in the epidermis layer becomes less efficient; also, diseases associated with intestinal malabsorption, such as celiac disease, Crohn’s disease, cystic fibrosis, ulcerative colitis, liver and kidney disorders and some pharmacological treatments, may contribute to the development of vitamin D deficiency [57].

Vitamin D deficiency is common in Italy too [52], in the geriatric population and during winter. Higher intakes may be required for obese subjects, due to the high depots of the vitamin in adipose tissue [58].

High amounts of vitamin D are contained in cod liver oil. Fish (especially fatty fish such as herring and salmon) are also major food sources, while pork liver, eggs, butter, high fat cheeses provide smaller amounts, but relevant to the total intake.

In the first stage of pregnancy, vitamin D (mainly Vitamin D3, the predominant form in the maternal blood) is involved in the regulation of cytokine metabolism and in the modulation of the immune system, thereby contributing to the embryo implantation and regulating the secretion of several hormones.

Vitamin D deficiency in mothers and breastfed infants was observed several decades ago in some Nordic countries, especially in winter, because of the lack of natural light [59]. However, vitamin D deficiency is very common during pregnancy even in countries with sunny climates and is associated with an increased risk of developing pre-eclampsia and gestational diabetes mellitus. The season of birth, ethnicity, and maternal prophylaxis during pregnancy affect the vitamin D status of infants. Low birth weight, impaired skeletal development, and respiratory infections and allergic diseases in the early years of life are often associated with inadequate contribution of vitamin D from the mother’s diet.

According to a recent systematic review, maternal supplementation during pregnancy reduces the risk of pre-eclampsia as well as preterm delivery and low birth weight [60].

Despite the lack of consensus on adequate intakes among different countries (Table 3), supplementation with vitamin D is recommended for all pregnant women at a dose of 600 IU/day (15 µg/day) [57].

In women at risk for vitamin D deficiency, the recommendations should be reach 1000–2000 IU/day. Prophylaxis with vitamin D should be planned from the beginning and throughout the pregnancy, as underlined also in the recent consensus document from the Italian pediatric societies [61].

Given the influence of sunlight exposure on vitamin D metabolism, attention to ethnic groups with hyper-pigmented skin or with little exposure to sunlight should be paid also during lactation. Moreover, the habitual dietary intake of vitamin D may be limited in specific conditions of higher requirements and in areas and/or countries with little availability of food sources [62]. Breastmilk, in fact, contains amounts of vitamin D (<80 IU/L) that are insufficient for deficit prevention in the first year of life [63]. An intake of 15 µg/day (600 IU/day), e.g., in women of childbearing age, is therefore needed to meet the requirement for vitamin D during breastfeeding, as highlighted in the aforementioned consensus document. These levels can be increased up to 1000–2000 IU/day for the whole breastfeeding period in presence of risk factors for deficiency.

### 3.5. Folic Acid

Folates play a crucial role in many metabolic reactions such as the biosynthesis of DNA and RNA, methylation of homocysteine to methionine, and amino acid metabolism. In fact, metabolically active forms of folates act as transport co-enzymes facilitating the transfer of carbon units from one compound to another. They are therefore essential for health: inadequate dietary levels can give rise to anemia, leucopenia, and thrombocytopenia [64]

Folates are mostly found in green leafy vegetables, fruits (such as oranges), cereals and offal. Their bioavailability from foods depends on the presence of anti-nutrients, which can reduce their absorption.

The requirement for folates undergoes a progressive increase throughout the periconceptional period, in association with the use for the development of cells and fetal tissues [65]. Maternal supplementation with folic acid is widely recommended to all women of childbearing age, especially to reduce the risk of neural tube defects [66,67]. According to recent studies, folic acid supplementation during pregnancy should also reduce the risk of congenital heart disease and support proper development of the placenta [68].

The RDA during pregnancy increases by 50% for pregnant as compared with non-pregnant women of childbearing age (600 µg/day vs. 400 µg/day). Ideally, supplementation should begin two months before conceiving and even reach 800 µg/day [69]. The use of folic acid-based supplements is considered as safe [65]. The benefits of higher amounts are unclear.

The folate concentrations in breastmilk increase progressively from colostrum to mature milk, reaching much higher levels than those measured in maternal plasma. The absence of a correlation between maternal status and breastmilk content suggests an active role of the mammary glands in the transport and regulation of folate secretion, only marginally influenced by dietary intakes [70].

Intakes of folates by the breastfeeding mother should be increased by 25%, up to 500 µg/day [71].

## 4. Focus on Conditions Associated with Nutrient Deficiencies

### 4.1. Smoking

The recommendation to abstain from smoking (including passive smoking) in pregnancy is mandatory, because of the increased risk of preterm delivery and low birth weight of the infant [72]. The negative effects of smoking can also extend beyond early childhood, as suggested by studies showing an association with the risk of overweight in infants born to smoking mothers [73]. A great deal of scientific evidence also supports the relationship between maternal smoking and respiratory diseases, especially in children exposed to smoke even in the prenatal period, among which a 20% increase in the incidence of asthma and bronchospasm was detected [74].

Smoking during pregnancy may also affect breastfeeding: cigarette smoke is associated with lower levels of DHA both in maternal plasma and in breastmilk, resulting in a reduced supply of this important fatty to the infant. In particular, DHA intake with milk by children born to smokers is reduced by 50% [31], almost in part due to a lowering effect on DHA synthesis in mammary gland cells [75].

### 4.2. Exclusion Diets

In order to reduce the risk of major nutritional deficiencies, attention should be paid during pregnancy and breastfeeding to women who exclude whole categories of foods from their diet, both for health or ethical reasons. The elimination from the diet of healthy mothers even of allergenic foods (soy, cow’s milk, eggs, peanuts, fish and shellfish) has, in fact, proved to be ineffective to prevent infant allergies [4]. In the absence of a diagnosis of celiac disease or gluten sensitivity, a gluten-free diet is not appropriate for healthy pregnant and lactating women; rather it can increase the risk of micronutrient deficiencies (folate, calcium, iron and zinc) more often in those who exclude gluten containing cereals [76,77].

Vegetarian (lacto-ovo vegetarian, pesco-vegetarian, etc.) and vegan diets are becoming increasingly prevalent in the general population. In particular, diets devoid of any food of animal origin, including eggs and milk should be attentively considered during pregnancy and lactation, even though their general nutritional characteristics include lower energy intake and protein content, higher supply of unsaturated fats and fiber in comparison with Western diets [78]. The inclusion of milk and eggs is associated with the adequate supply of nutrients to pregnant women, while the exclusion of all animal foods makes the vegan diet likely inadequate in this life period, if not properly integrated. A systematic review of the literature highlighted an increased risk of iron and vitamin B 12 deficiency and low birth weight in association with vegetarian or strictly vegan diets, despite the absence of adverse effects for the fetus [79].

Calcium deficiency should also be considered in diets excluding dairy, in absence of appropriate replacement, due to the higher availability with foodstuffs of animal origin [50].

The lack of food sources of omega-3 fatty acids in the gestational period and in the weeks after childbirth should be properly monitored and corrected, in order to reach the intakes recommended in order to maintain the health status of the mother and to ensure the correct neuromotor and visual development to the offspring [27].

Another increasing issue, concerning in particular low-income populations, is the low consumption of fruits and vegetables (in absence of fortified products), associated with insufficient intakes of specific micronutrients [80]. Supplementation with a mix of vitamins and minerals effectively reduces the risk of preterm delivery and infants of low weight at birth in developing countries, especially in women with a high body mass index [81].

During lactation, exclusion diets require careful control. In fact, even though the macronutrient composition of human milk is relatively stable and reproducible [82], its micronutrient content seems to be significantly dependent upon the maternal diet [83].

### 4.3. Obesity

The high prevalence of obesity in the general population, and therefore in women of childbearing age, increases the risk of complications in pregnancy as well as of infants with high weight at birth and predisposed to obesity in early life, as recognised by the WHO. Weight loss before conception is effective in reducing the risk of very high or very low birth weight and increasing infant survival [84].

An excessive weight gain during pregnancy leads to morphological alteration of the placenta, due to the high degree of systemic inflammation associated with poor nutritional status, as observed in Italian overweight women [85].

However, even in obese pregnant women the caloric intake should not fall below 1600 kcal/day, with an adequate supply of nutrients, especially protein and calcium [86]. This is more important during lactation, when the energy intake should be sufficient to reach a healthier weight and to compensate for the energy expenditure associated with galactopoiesis.

### 4.4. Previous Bariatric Surgery

Reserved to cases of severe obesity, bariatric surgery induces malabsorption (of both macronutrients and micronutrients) and can lead to prolonged deficits. In cases of post-surgery conception, specific nutritional deficiencies can affect visual acuity (vitamin A) and the development of the nervous system (vitamin B12) and neural tube (folates) [87,88].

Even if the risk of gestational diabetes and macrosomia at birth may be reduced in obese women previously undergone bariatric surgery, perinatal mortality rate and preterm delivery significantly increase, due to the post-surgical complications [89].

No differences have been reported in the literature on the composition of breastmilk from women who underwent bariatric surgery.

### 4.5. Adolescence

To date, there is limited data on the precise nutritional needs of adolescents during pregnancy and lactation, but the increased risk of preterm delivery and low birth weight has been correlated to alterations of placental function in pregnant adolescents [90,91]. It has been shown that, in addition to biological immaturity, the inadequate nutritional status (e.g., breakfast skipping, improper weight loss diet, energy-dense foods) could play important roles in the onset of complications in the perinatal period [92].

Even with adequate weight gain in the early months of pregnancy, teenagers do not seem to properly mobilize their fat storage during the prenatal period, which is necessary to maintain optimal fetal growth. This is a kind of “nutritional competition” with the fetus, probably related to rapid growth and hormonal changes associated with an increased requirement for macro and micronutrients [93].

Folic acid, iron, zinc, calcium, vitamin C, and vitamin D (in addition to energy and protein), are the main micronutrients for which the requirement physiologically increases during pregnancy and lactation. Moreover, the prevention of nutrient deficiencies (also by using supplements) is often overlooked, due to the lack of knowledge or unplanned pregnancy [92].

### 4.6. Multiple and Repeated Pregnancies

A careful assessment of the nutritional status, aimed at optimizing the dietary intake of micronutrients and at planning fortification, is particularly indicated for women who experience repeated pregnancies, especially in case of multiple pregnancies separated by short time periods, which increase the risk of depletion of maternal reserves [94]. Some studies have reported an increased risk of sideropenic anemia in mothers and children up to six months of life, in association with low hemoglobin levels in the first two trimesters of pregnancy. Again, some evidences support the benefits of supplementations with calcium, magnesium, zinc, multivitamins, and essential fatty acids.

As far as repeated pregnancies are concerned, special attention should also be paid to the maternal weight between gestations [95].

Breastfeeding should be recommended even at the onset of a new pregnancy, particularly during the first and second trimester. Even in the absence of equally strong evidence, the Italian Society of Perinatal Medicine (SIMP) underlines the sustainability of breastfeeding even in the third trimester of pregnancy [96].

### 4.7. Previous Pregnancy-Related Complications

Women with a previous history of multiple births, preterm delivery and pre-eclampsia should be evaluated as concerns their diet and specifically the intake of micronutrients that can reduce the risk of preterm delivery, pre-eclampsia, and intrauterine growth restriction in subsequent pregnancies [97]. According to a Swedish study, consumption of a diet rich in fruits, vegetables, whole grains and fish and with adequate hydration in the early months of pregnancy correlates with a reduced risk of preterm delivery, with benefits higher than those obtained by excluding less healthy foods from the maternal diet [98]. These observations are partly confirmed by the Dutch Generation R study, showing that the degree of adherence to the Mediterranean diet is positively associated with maternal serum levels of folic acid and vitamin B12 and that, on the other hand, a poor diet correlates with increased risk of hypertension in the gestational period [99]. Some observations also support the efficacy of supplementation with multivitamins and multiminerals [100,101].

## 5. Conclusions

Very hard scientific evidence supports the importance of lifestyle and dietary habits (with adequate micronutrient intakes) during pregnancy and breastfeeding, for the health status of women and their offspring.

The consumption of a varied and balanced diet from the preconceptional period is essential to ensure maternal well-being and favorable outcomes of pregnancy. Even in the most industrialized countries, specific dietary intakes in pregnancy and lactation are often inadequate. With respect to the Italian population, the available data indicate that intakes of selected nutrients are often insufficient by both selected population groups and by pregnant and lactating women. This particularly applies to DHA, iron, iodine, calcium, folic acid, and vitamin D (Table 4).

Notably, the values recorded in Italy [25,53] differ from those recommended by the Italian guidelines [7] from 40% (iodine) to 85% (vitamin D).

In particular:

- Folic acid: there is need to supplement maternal diets during pregnancy, through food fortification (within proper diets) and via the use of supplements in the preconceptional period, in agreement with national and international guidelines.
- Vitamin D: there is no homogeneous consensus on its recommended intakes [98]; in Italy, a recent consensus document published by the Societies of Pediatrics emphasizes the high prevalence of deficiency and the importance of prophylaxis also during pregnancy and breastfeeding.
- Iron: even though there is a general agreement on the benefits of systematic supplementation in populations at high-risk of anemia during pregnancy, different countries provide different recommendations; in general, iron supplementation should be decided on the basis of individual clinical assessment.
- Iodine: adequate intakes must be ensured throughout pregnancy, e.g., by using foods rich in iodine and iodized salt.
- Calcium: a large proportion of the European (and Italian) fertile female population do not reach optimal values; moreover, particular attention should be paid to its bioavailability from different foods.
- DHA: benefits during pregnancy and lactation are confirmed by the most recent studies; inadequate intakes are associated with low consumption of fish rich in omega-3.

From a nutritional point of view, particular attention should be paid to women of childbearing age following exclusion diets, especially during pregnancy and lactation, due to the increased risk of not reaching the adequate supply of nutrients to support maternal and infant health.

Specific cases requiring clinical examinations and targeted interventions in the perinatal period include women with weight problems (underweight or overweight/obese), smokers, adolescents, mothers who have had multiple or close pregnancies, and those with previous unfavorable pregnancy outcomes.

## Figures and Tables

**Table 1:** Content of EPA + DHA and individual EPA and DHA in different fishery products [23].

Foods	EPA + DHA (g/100g)	EPA (g/100g)	DHA (g/100g)
Salmon	1.95	1.01	0.94
Herring	1.66	0.97	0.69
Anchovy	1.45	0.54	0.91
Mackerel	1.30	0.90	1.40
Trout	0.73	0.20	0.53
Swordfish	0.76	0.11	0.65
Sea bass	0.60	0.24	0.36
Squid	0.49	0.15	0.34
Mussel	0.44	0.19	0.25
Sole	0.25	0.14	0.11
Cod	0.18	0.06	0.12

**Table 2:** Different recommended intakes for iron in pregnancy and breastfeeding. Modified from [41].

Country/Institution	Pregnancy (mg/day)	Breastfeeding (mg/day)
Italy [6]	27	11
Germany-Austria-Switzerland	30	20
Nordic Countries	-	15
WHO/FAO ^1^	-	10–30 ^2^
France	30	10
Institute of Medicine	27	9
Scientific Committee on Food	-	10
The Netherlands	11-15-19 ^3^	20

^1^ Supplementation recommended to all pregnant women; ^2^ According to bioavailability; ^3^ In the 1^st^, 2^nd^ and 3^rd^ trimester respectively.

**Table 3:** Recommended intakes for vitamin D in adult and elderly population, and in pregnant and breastfeeding women, in some European countries. Modified from [57].

Countries	Adult (μg/die)	Elderly (μg/die)	Pregnancy (μg/die)	Breastfeeding (μg/die)
Austria	20	20	20	20
Belgium	10–15	15	20	20
France	5	5	10	10
Germany	20	20	20	20
Ireland	0–10	10	10	10
Italy	15	20	15	15
Spain	15	20	15	15
Switzerland	20	20	20	20
Turkey	10	10		
The Netherland	10	20	10	10
Scandinavian Countries	10	20	10	10
UK	–	10	10	10

**Table 4:** Recorded daily intakes of selected micronutrients in the Italian adult population and Population Reference Intakes (PRI) in pregnancy and breastfeeding, according to Italian RDA [7].

Reference	Nutrient	Adult (Intake)	Pregnancy (PRI)	Breastfeeding (PRI)
[25]	Omega-3 (+DHA)	170 mg	250 mg (+ 100–200 mg)	250 mg (+ 100–200 mg)
[53]	Iron	10.4 mg	27 mg	11 mg
[7]	Iodine	85–88 µg	200 µg (AI) ^1^	200 µg (AI) ^1^
[53]	Calcium	730 mg	1200 mg	1000 mg
[53]	Folic acid	305 µg	600 µg	500 µg
[53]	Vitamin D	2.3 µg	15 µg	15 µg

^1^ AI: adequate Intake.
